# Correction: Schlösser et al. Anti-HIV-1 Effect of the Fluoroquinolone Enoxacin and Modulation of Pro-Viral hsa-miR-132 Processing in CEM-SS Cells. *Non-Coding RNA* 2025, *11*, 8

**DOI:** 10.3390/ncrna11030040

**Published:** 2025-05-16

**Authors:** Verena Schlösser, Helen Louise Lightfoot, Christine Leemann, Seyedeh Elnaz Banijamali, Aathma Merin Bejoy, Shashank Tiwari, Jeffrey L. Schloßhauer, Valentina Vongrad, Andreas Brunschweiger, Jonathan Hall, Karin J. Metzner, Jochen Imig

**Affiliations:** 1 Institute of Pharmaceutical Sciences, ETH Zurich, 8093 Zurich, Switzerland; 2 Division of Infectious Diseases and Hospital Epidemiology, University Hospital Zurich, University of Zurich, 8091 Zurich, Switzerland; 3 Institute of Medical Virology, University of Zurich, 8057 Zurich, Switzerland; 4 Max Planck Institute of Molecular Physiology, Chemical Genomics Centre, 44227 Dortmund, Germany; 5 Department of Pharmaceutical and Medicinal Chemistry, University Würzburg, 97074 Würzburg, Germany

Seyedeh Elnaz Banijamali was not included as an author in the original publication [1]. The corrected Author Contributions statement appears here: Seyedeh Elnaz Banijamali executed experiments and analyzed data related to the SHAPE-MaP assay and read and approved the final manuscript.

The authors state that the scientific conclusions are unaffected. This correction was approved by the Academic Editor. The original publication has also been updated.
