# Supplementary material for: Anti-HIV-1 Effect of the Fluoroquinolone Enoxacin and Modulation of Pro-Viral hsa-miR-132 Processing in CEM-SS Cells
Source: Noncoding RNA. 2025 Jan 20;11(1):8. doi: 10.3390/ncrna11010008 (PMC11755467; doi:10.3390/ncrna11010008)

Figure S1: CEM-SS Enoxacin effect and qRT-PCR

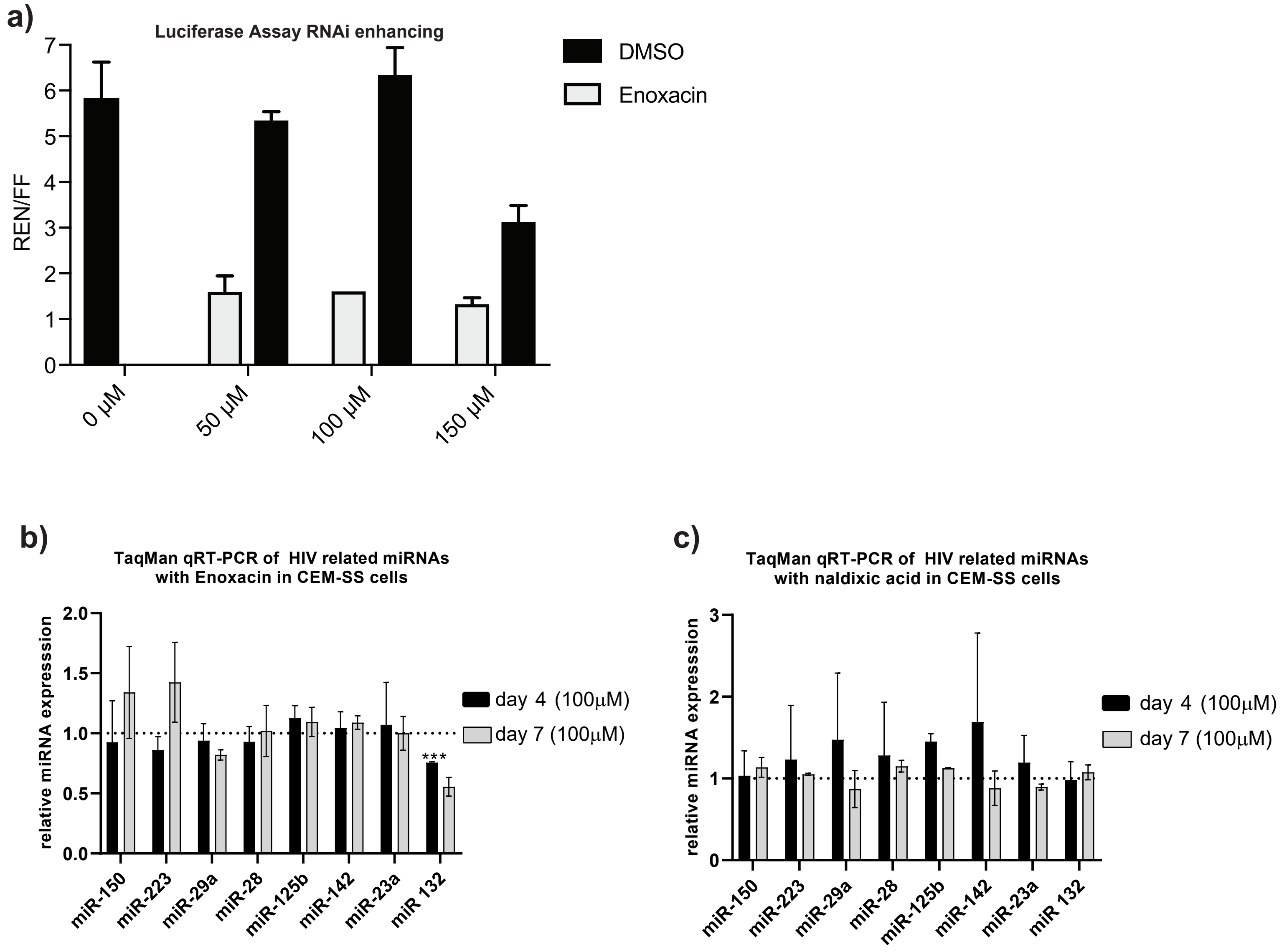

Figure S2: Enoaxcin and Nalidixic acid p24 ELISA

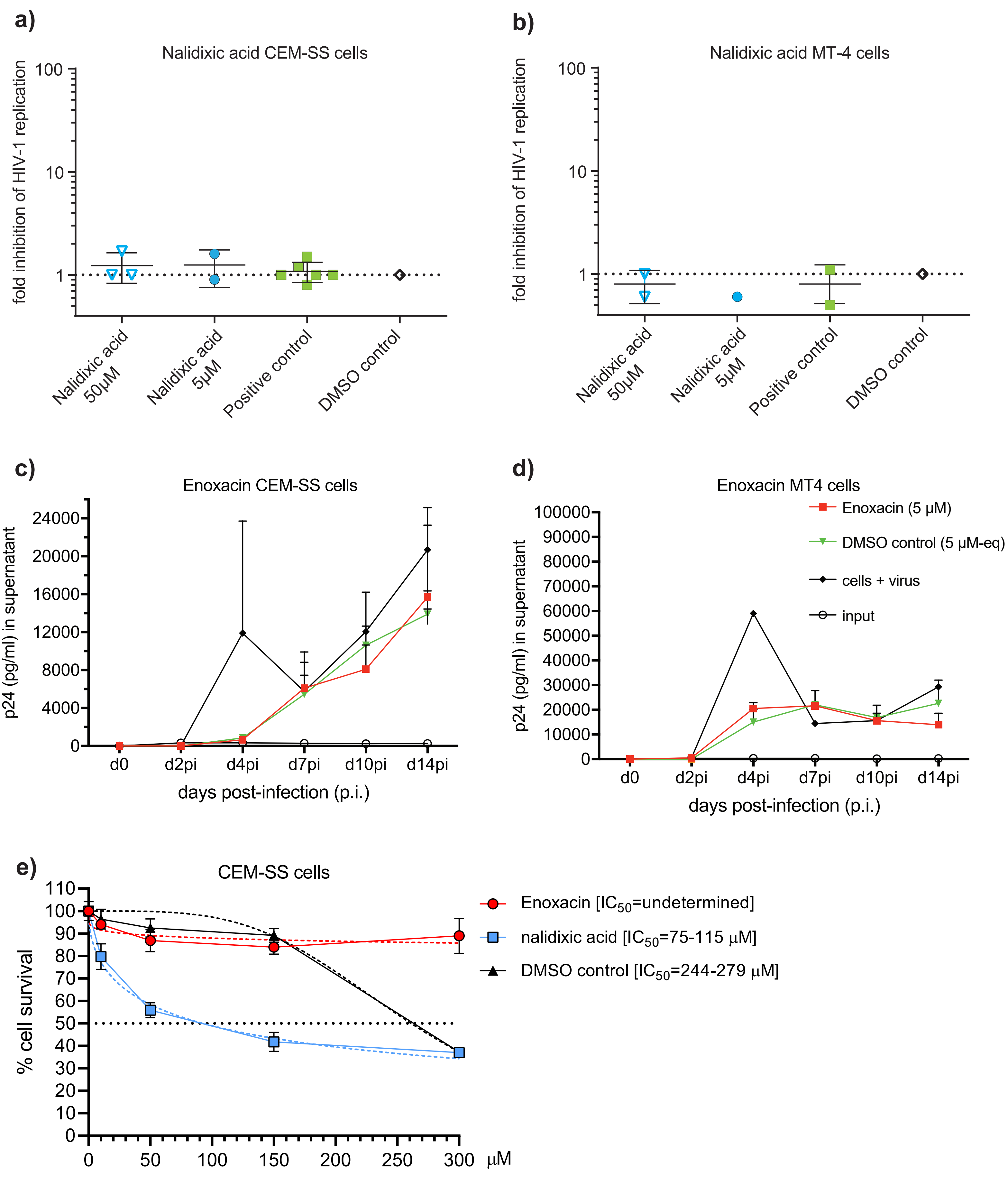

Figure S3: FACS CEM-SS and MT-4 transfection efficacy

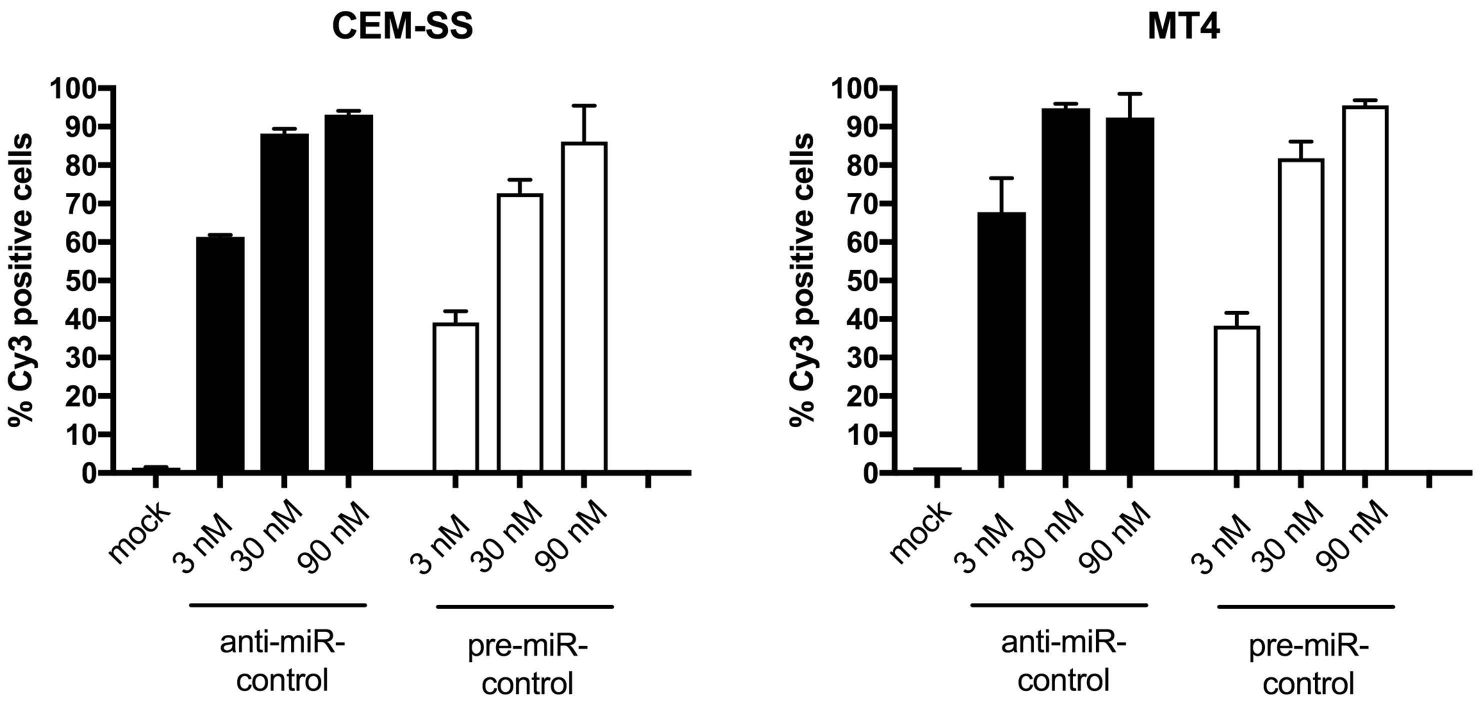

Figure S4: uncropped 5p Dicer assays and individual quantifications

a) Dicer-1 assay: miR-132-5p (Rep 1)

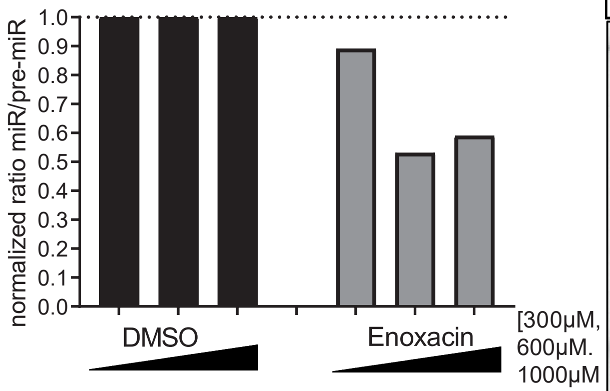

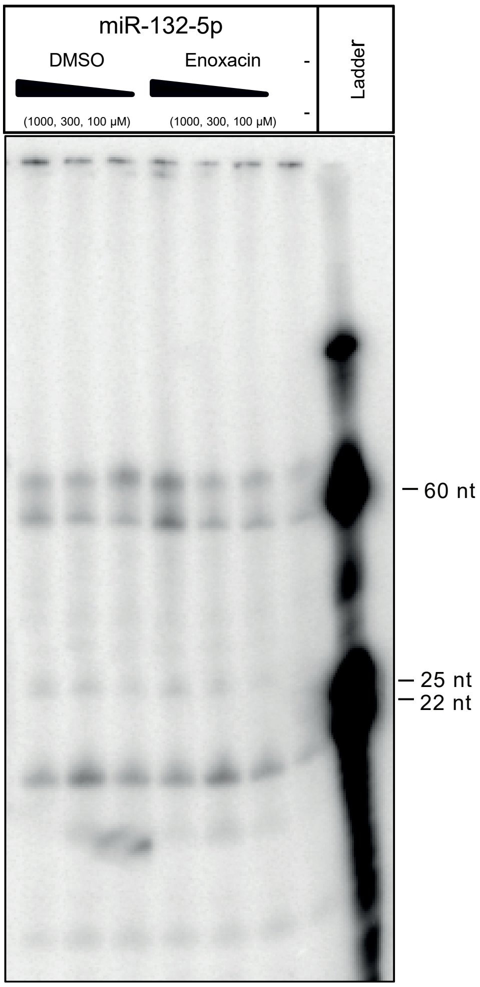

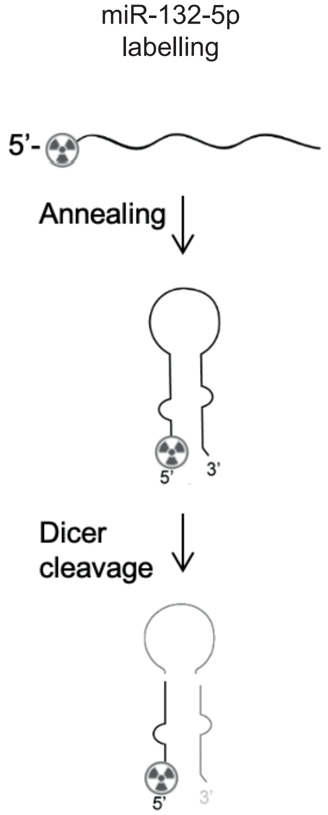

Dicer-1 assay: miR-132-5p (Rep 2)

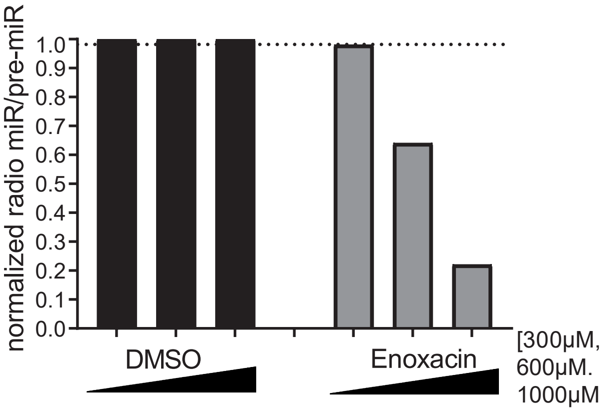

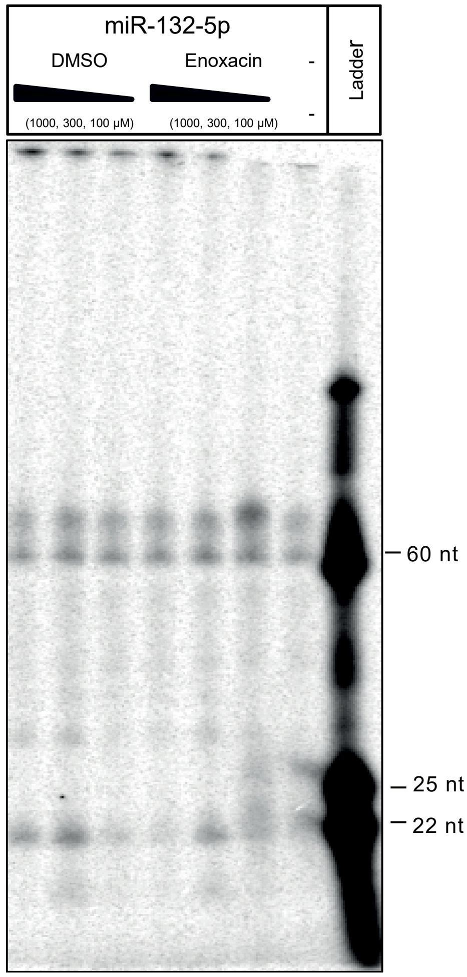

Figure S4: uncropped 3p Dicer assays and individual quantifications

b)

Dicer-1 assay: miR-132-3p (Rep 1)

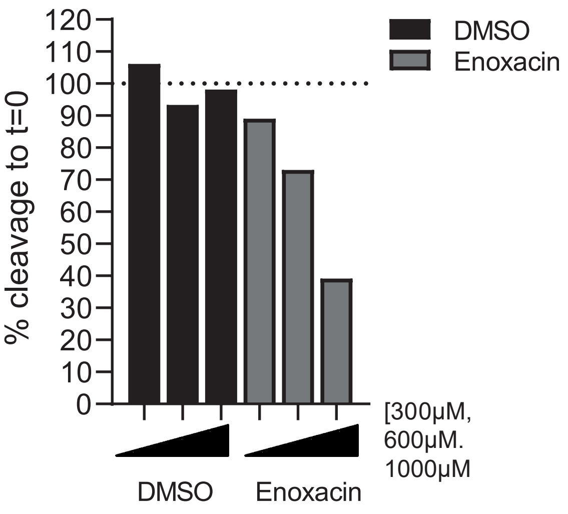

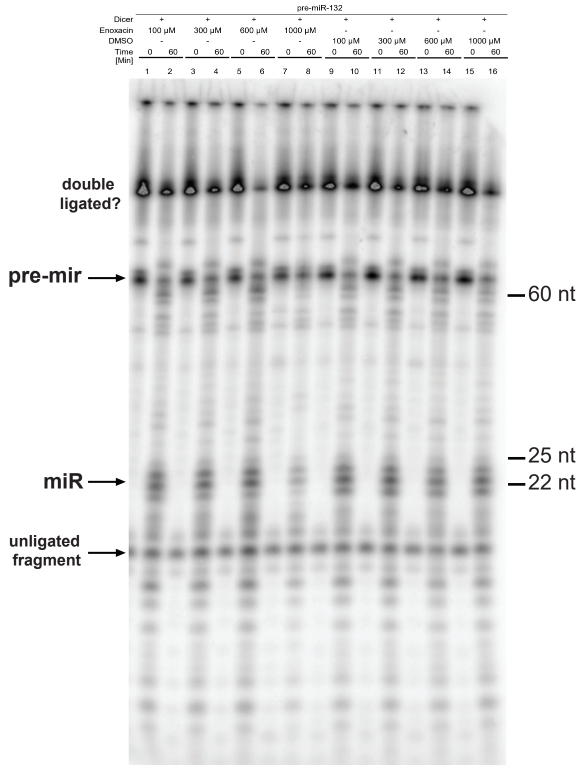

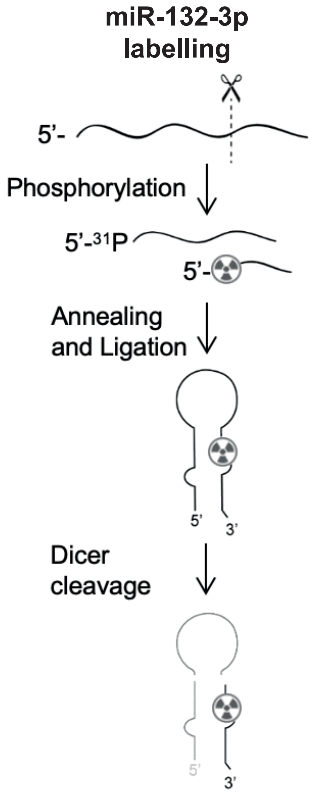

Dicer-1 assay: miR-132-3p (Rep 2)

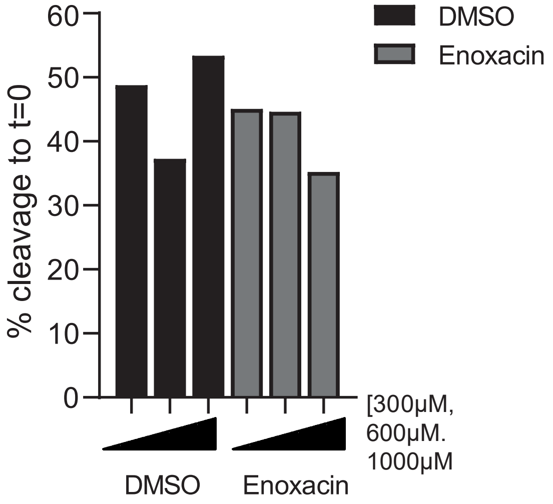

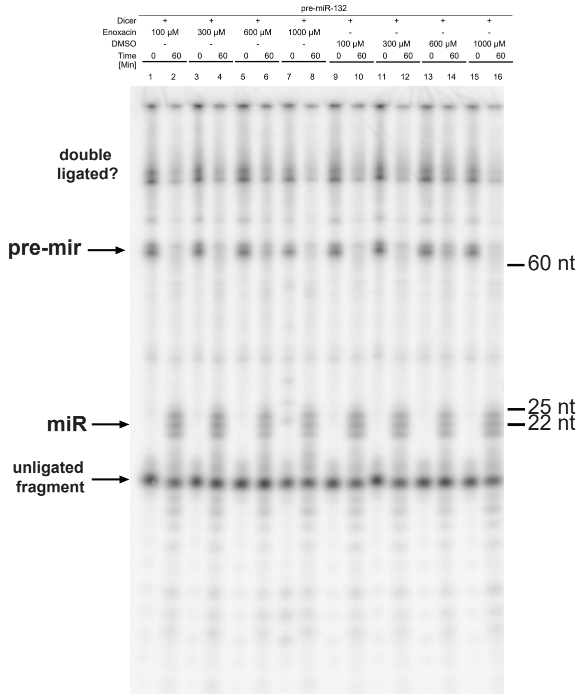

Figure S5: SHAPE reactivity plots pre-miR-132

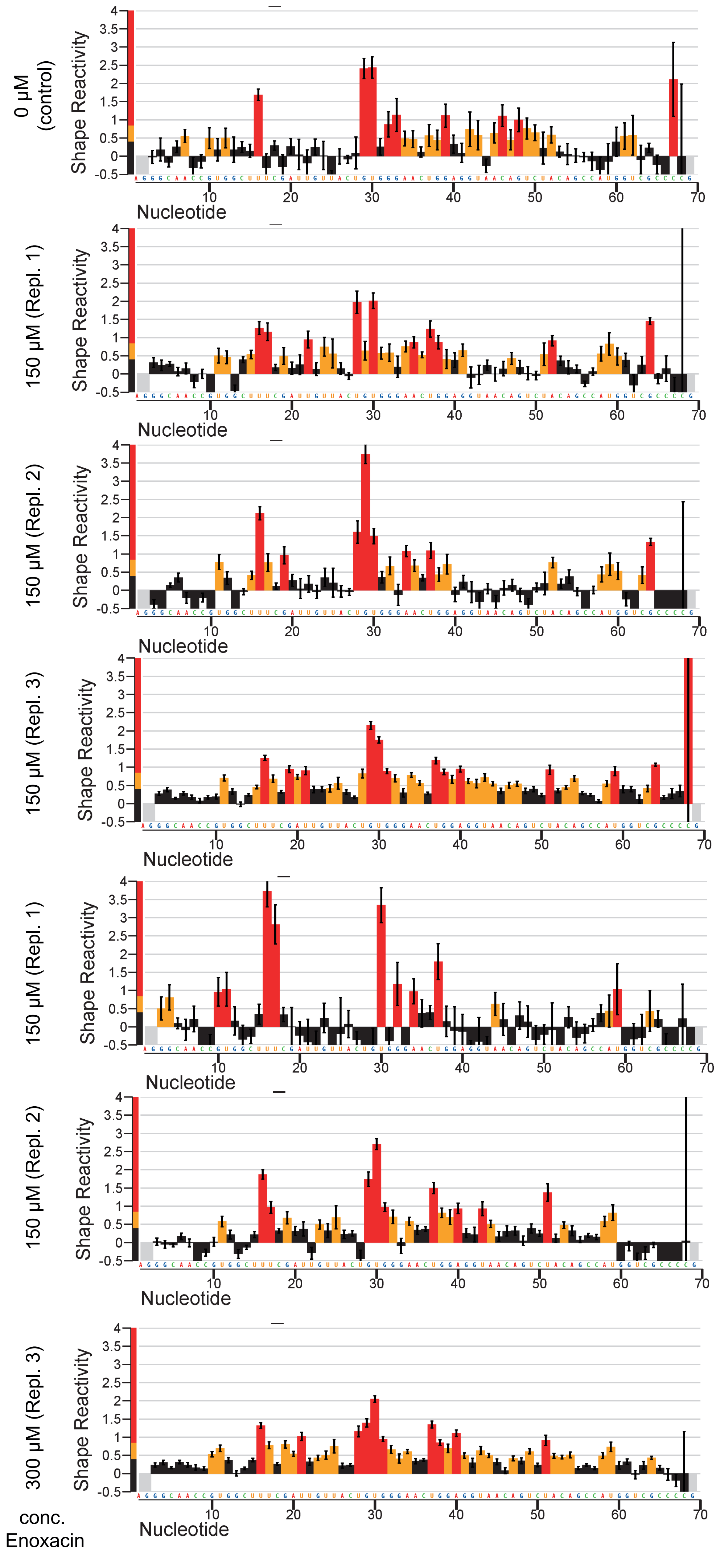

Supplement: Supplementary file 1 [file ncrna-11-00008-s001.zip › Fig S1-5.pdf]
