# Supplementary material for: Anti-HIV-1 Effect of the Fluoroquinolone Enoxacin and Modulation of Pro-Viral hsa-miR-132 Processing in CEM-SS Cells
Source: Noncoding RNA. 2025 Jan 20;11(1):8. doi: 10.3390/ncrna11010008 (PMC11755467; doi:10.3390/ncrna11010008)
Supplement: Supplementary file 1 [file ncrna-11-00008-s001.zip › Tables.docx]

Table S1: DNA template for pri-miR 132 and the primers used for PCR assembly

| Name | Sequence (5’->3’) |
| --- | --- |
| DNA template for pri-miR-132 | *TTCTAATACGACTCACTATA*GGGAGACCGCCCCCGCGTCTCCAGGGCAACCGTGGCTTTCGATTGTTACTGTGGGAACTGGAGGTAACAGTCTACAGCCATGGTCGCCCCGCAGCACGCCCACGCGC |
| F1-PCR assembly | TTCTAATACGACTCACTATAGGGAGACCGCCCCCGCGTCTCCAGGGCAACCGTGGCTT |
| R2-PCR assembly | ACCTCCAGTTCCCACAGTAACAATCGAAAGCCACGGTTGCCCTG |
| F3-PCR assembly | TGTTACTGTGGGAACTGGAGGTAACAGTCTACAGCCATGGTCGCCCCGCA |
| R4-PCR assembly | GCGCGTGGGCGTGCTGCGGGGCGACCA |

T7 prompter sequence in DNA template in italic. In red additional nucleotides added to increase the transcription.

Table S2: Sequence of pre-miR132 used for chemical probing and the primers used in primer extension and mutational profiling.

| Name | Sequence (5’->3’) |
| --- | --- |
| Pre-miRNA 132 | GGGAGACCGCCCCCGCGUCUCCAGGGCA**ACCGUGGCUUUCGAUUGUUACUGUGGGAACUGGAGGUAACAGUCUACAGCCAUGGUC**GCCCCGCAGCACGCCCACGCGC |
| RT-primer | GCGCGTGGGCGTGCTGCGGG |
| Fw-PCR-Step1 | GACTGGAGTTCAGACGTGTGCTCTTCCGATCTTAATGGGAGACCGCCCCCGCGTCT |
| Re-PCR-Step1 | CCCTACACGACGCTCTTCCGATCTCAATGCGCGTGGGCGTGCTGCGGG |
| Fw-PCR-Step2 | CAAGCAGAAGACGGCATACGAGAT*NNNNNN*GTGACTGGAGTTCAGAC |
| Re-PCR-Step2 | AATGATACGGCGACCACCGAGATCTACACTCTTTCCCTACACGACGCTCTTCCG |
